# Entropy, or Information, Unifies Ecology and Evolution and Beyond

**DOI:** 10.3390/e20100727

**Published:** 2018-09-21

**Authors:** William Bruce Sherwin

**Affiliations:** Evolution & Ecology Research Center, School of Biological Earth and Environmental Science, UNSW Sydney, Sydney 2052, Australia; W.Sherwin@unsw.edu.au; Tel.: +61-(0)2-9385-2119

**Keywords:** Shannon, diversity-profile, entropy, selection, linkage-disequilibrium, gene-expression, evolutionary algorithms, mutual information, adaptation, artificial intelligence

## Abstract

This article discusses how entropy/information methods are well-suited to analyzing and forecasting the four processes of innovation, transmission, movement, and adaptation, which are the common basis to ecology and evolution. Macroecologists study assemblages of differing species, whereas micro-evolutionary biologists study variants of heritable information within species, such as DNA and epigenetic modifications. These two different modes of variation are both driven by the same four basic processes, but approaches to these processes sometimes differ considerably. For example, macroecology often documents patterns without modeling underlying processes, with some notable exceptions. On the other hand, evolutionary biologists have a long history of deriving and testing mathematical genetic forecasts, previously focusing on entropies such as heterozygosity. Macroecology calls this Gini–Simpson, and has borrowed the genetic predictions, but sometimes this measure has shortcomings. Therefore it is important to note that predictive equations have now been derived for molecular diversity based on Shannon entropy and mutual information. As a result, we can now forecast all major types of entropy/information, creating a general predictive approach for the four basic processes in ecology and evolution. Additionally, the use of these methods will allow seamless integration with other studies such as the physical environment, and may even extend to assisting with evolutionary algorithms.

## 1. A Shared Basis for Ecology and Evolution

Ecology and evolution are often studied separately, with researchers focusing only on a single aspect of information or entropy: molecular variation, species variation, etc. All of these aspects of information can be seen in a larger, unified framework with nested levels such as molecules, individuals, populations, species, and ecosystems. Each of these information types manifests four common features [1]:

- Innovation (e.g., mutation, recombination, divergence and speciation, behavioral innovation)
- Transmission and replication (e.g., inheritance)
- Movement (e.g., migration, pollen dispersal, etc.)
- Adaptation (e.g., selection, behavioral avoidance of harm)

Often the same level of organization will incorporate several competing or cooperating methods of innovation, transmission, movement, and adaptation. 

There are many types of information, but for simplicity, this article will focus largely on two analogous types of information: alternative species in ecological assemblages and DNA alternatives in one species (‘alleles’). Additionally, within those two types, discussion will mostly be restricted to binary cases, such as presence or absence of two alternative species in an assemblage, or presence or absence of two alternative ‘nucleotides’ in DNA e.g.,


…AC**A**GCCT…
vs.
…AC**T**GCCT…

These alternatives or ‘alleles’ can be characterized by the probabilities P(**T**) and P(**A**) in the biological population (usually at any position in the DNA, called a ‘SNP’ or single nucleotide polymorphism, only two of the possible four nucleotides are found). These molecular variants are exactly analogous to alternative species in ecological assemblages, and in most cases, measures or forecasts made in one of these areas have been, or could be, transferred directly to the other.

This article will discuss how entropy/information methods are well suited to analyzing and forecasting the four common processes of innovation, transmission, movement, and adaptation in ecology and evolution. Despite the focus on a few variant types, this will apply broadly to variants of all types: DNA, epigenetics, behavior, species, the physical environment, etc., as well as their interactions [2,3].

## 2. Background: Measuring Biological Entropy, Information and Diversity

**Figure 1:**
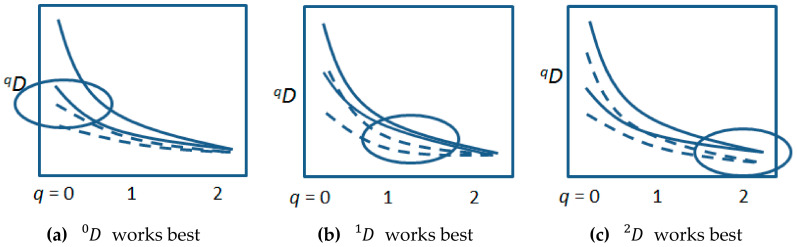
Confidence limits for Dq values for two hypothetical localities, one locality shown as a pair of solid lines, the other locality shown as a pair of dashed lines (the mean curves would be between the two confidence limits, but are omitted for clarity). The circled areas in each of the three panels show cases where discrimination between the assemblages of species or genes at the two localities is more clearly identified by (**a**) *q =* 0, (**b**) *q =* 1, or (**c**) *q =* 2, respectively.

Measurement of ecological or evolutionary variants uses various entropy or information measures (Table 1a). The measures are all part of a ‘*q*-profile’ derived from a general power-sum of variant proportions (0≤q≤∞) [4,5]), composed of Dq measures on a common scale of the ‘effective’ number of variants, which means the number of equally-frequent variants that would give the same entropy (Hq) as the typically unequal array of variants in the sampled system. The use of the Dq profile has been recommended because each *q* value emphasizes different aspects of the diversity [6,7]; for example, higher *q* values emphasize the more common variants [5]. These different sensitivities mean that for different biological cases, different parts of a Dq profile might provide the best discrimination (Figure 1). A comparison of *q* = 0, 1, 2 shows similar results in 85% of studies, and where one measure is better, there is a clear explanation for this [5]. For example, in a study of invasive mosquito populations *Aedes j. japonicas*, *q =* 1 was more sensitive than *q =* 2 for tracing invasion patterns [8], presumably because *q =* 2 emphasizes common variants, rather than the rare variants that tend to be lost during periods of small population size at successive newly invaded sites.

Of course, integration of ecology and evolution would be easiest if they used the same measures of information/entropy. Table 1 shows that they do use the same measures, but with different emphasis. This article proposes that although the entire *q*-profile is useful, *q =* 1 based on Shannon information/entropy is uniquely informative, combining many important properties for measurement of diversity within and between groups [28,29]. This combination of properties has led to *q =* 1 becoming very frequently used in ecology (Table 1a). Other measures have some of these properties, but not all [5]. First, Shannon’s sampling can be adequately adjusted by modern methods to account for the possibility of missing rare types, whereas the same problem for *q =* 0 is not completely correctable [9,10], and leads to the wide confidence limits for *q =* 0, seen in Figure 1. Second, within-group D1 increases linearly with pooling of equally-diverse, completely distinct groups, which does not happen with some other measures.

Third, we must deal not only with the entropy or diversity within a single system (α), but also entropy or diversity due to divergence or differentiation between systems (β). Extensive equations for α- and β-diversity with all levels of *q* are in the supplements of a past review [5]. For β-differentiation between localities, *q =* 1 measures show strict monotonicity, always increasing with increasing differentiation between groups of molecules or species, whereas *q =* 2 measures do not always do this [30]. In particular, there is no *q =* 2 β-measure that creates complete independence between α (within-group), β (between-group), and γ (total) diversity [5]. This contrasts with *q =* 1 measures that are based on Shannon’s explicitly hierarchical theory, and thus always ensure complete independence of α and β. There are various fixes for the problems of *q =* 2 β-measures [21,22], but it is better to realize that such measures have properties that, while useful, do not always reflect differentiation between groups [31]. 

## 3. Forecasting Biological Entropy, Information, and Diversity, Based on the Four Processes Common to Ecology and Evolution

Predictions under different hypothetical biological scenarios can be tested by measurement—the key to scientific advancement. Thus we need to make forecasts of the expected value under specified histories of the four processes: innovation, transmission, movement, and adaptation. Testing for agreement with, or departure from, those predictions allows us to infer the likely underlying processes. In this article, there is emphasis on forecasts based on algebraic modeling of the underlying processes, rather than from curve-fitting, because of the understanding of the system that can be achieved from algebraic expressions. The predictive theory for entropy/information *q* = 1, is already sufficiently complete to be used, together with predictions for other values of *q*, to unite analysis of all aspects of ecology and evolution.

Table 1b shows that there is a huge body of predictive theory for *q =* 2 measures in evolution (some also transferred to ecology), but that as late as 2006, we still had little predictive power for *q =* 1 (Shannon), despite some early attempts [5,32]. Since that time, we now have *q =* 1 & 2 predictions for a wide range of situations involving the four basic information processes—Innovation, Transmission, Movement, and Adaptation (Table 2). In some cases *q =* 1 methods outperform those based on other values of *q*, mainly because the *q =* 1 methods are completely additive, and robust to a very wide range of population sizes, dispersal rates, and mutation modes ([33] and supplement 2 of [5]). Nevertheless, it can also be seen that there are still areas where further research is needed for *q =* 1, labelled ‘Not Yet’ in Table 2.

Table 2 shows that innovation of new variants can take various forms, which can be dealt with by entropic methods just as well as by other methods. For variation within species, DNA mutation can take at least three different forms (Table 2) each yielding its own mathematical expressions; often all forms might occur on a single DNA molecule [19,20]. SNP mutation is focused on a single ‘nucleotide’ in the DNA sequence, showing forward (and possibly back) mutation to create variant ‘alleles’. SNP innovation is extremely rare (~10^−9^ per generation), but because most genomes contain billions of nucleotides, and many species have persisted for a huge number of generations, SNPs have become ubiquitous in natural populations. IAM is an extreme alternative innovation mechanism, used when we consider a long DNA sequence such as a thousand-nucleotide protein-coding region. In this case, mutations usually create a sequence that has never occurred before, so this is called the ‘infinite alleles model’ (IAM), which has its own mathematical formulation. Finally, SMM is another type of mutation model called ‘stepwise’, in which new variants progress through adjacent functionally similar states, such as proteins mutating by a single unit of net surface charge, yielding variants of 2− 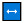 1− 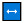 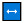 0 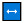 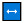 1+ 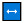 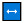 2+, etc. Nowadays this model is also used to approximate innovation in repetitive DNA regions (e.g., ‘microsatellite’ fingerprint DNA). Each model—SNP, IAM, and SMM—is only an approximation, and there are other innovation processes such as insertion or deletion of nucleotides, rearrangements, and epigenetic modification.

What about ecological innovation? Of course, this is ultimately highly reliant on genetic innovation, however for modeling at the ecological level, some of the mutation models (SNP/IAM) have also been employed as approximations for the production of novel species [18]. There is a wide range of speciation types [40], so a wide range of models are needed. For example, speciation that occurs by the alteration of a single character, such as the ‘magic traits’ discussed in the speciation literature [41], could be modeled by SNP, or by SMMif the novel species have an ordered relationship (e.g., gradual addition of more gill-rakers in a series of fish speciation events). On the other hand, the IAM might be more appropriate for speciation occurring via relatively rapid (but not instantaneous) multiple changes, a factor that has recently been added to the “neutral” theory of biodiversity [42]. These multiple changes can occur completely simultaneously by processes such as gross chromosomal alteration affecting many characters at once, due to entire genes being duplicated, deleted, or rearranged into a novel linear order, which affects their expression (called ‘position effect’). On the other hand, the multiple changes might accumulate during a period when two parts of a single species’ range are separated by a barrier that appears then later disappears, such as a sea-level rise inundating the center of the species’ range for 10,000 years, then receding. This can be modeled as a continuous process [42], or might be modeled as IAM where each new species is regarded as a totally novel variant, based on myriad genetic differences, occurring during the relatively short period of separation. Whatever the innovation mechanism assumed, Table 1b and Table 2 show that there are entropic forecasts available.

The other major method of innovation is through the breakage of associations between different variants, such as an association of high dispersal ability with low reproduction. At the molecular level, this is called ‘recombination’: the exchange of information by physical breakage and reunion of the DNA string of information, to unite SNP variants that were previously on separate DNA molecules (or ‘haplotypes’), such as,

…AC**A**GC**C**T…     …AC**A**GC**G**T… *and*    →    *and*
…AC**T**GC**G**T…    …AC**T**GC**C**T…

Of course, innovation by recombination is limited by the availability of variants that originate from mutation; However, given that many such variants are available, recombination produces new combinations at a vastly faster rate than the original mutation, giving recombination huge importance in evolutionary biology. A typical pair of SNP locations experiences 50% recombination per generation, in diploid individuals such as most higher organisms. Entropic methods are at the core of many modern methods to assess recombination [5], or rather the effect of low recombination rates to create ‘linkage’ into ‘multi-SNP haplotype’ molecules, which may have great adaptive significance [17]. The ecological parallel to linkage is correlation of phenotypic traits (actually often due to genetic linkage), and innovation occurs when these correlations occur, or break down, due to chance or adaptive processes discussed below.

Transmission of information is also extremely well-analyzed by entropic methods. The second row of Table 2 shows the modeling of stochastic transmission of several types of variant in finite populations, whose equations have also been applied to transmission of members of different species in ecological assemblages [18]. Simple replication modes, as seen with cells in bodies, or individuals in an ecosystem, have an exponential rate equation (or a ‘logistic’ equation when restricted by resources etc.) which can be expressed in entropic terms [43]. Other replication modes are discussed in the next section.

Movement of variants (e.g., alternative alleles or members of alternative species) can also be assessed very well using entropic analysis [5]. Briefly, for any pair of locations, lower dispersal, smaller population size, or greater elapsed time since separation, will increase divergence between the arrays of variants (types of alleles, species, etc.). This divergence can be characterized as mutual information (*I*, *q =* 1) between variant identity and location of origin [5]. In other words, if there is less sharing of variants between locations, then knowing the type of an individual (i.e., what species it is or what allele it possesses) gives better information about that individual’s geographic origin. There is an inverse relationship between mutual information and effective dispersal rate, over a very wide range of population sizes and dispersal rates [5]. For genes, the *q =* 1 equations apply to a wide assortment of types of genetic variant, seen in the second row of Table 2, and can be used to estimate dispersal from genetic data, a task at which they can outperform other methods [5].

Of course, species can vary widely in their dispersal ability [44,45], and there is also considerable genetic variation of dispersal ability within a single species, such as wing-polymorphism [46]. Nevertheless, some authors have successfully forecast assemblages of species or allelic variants, based on the assumption that any variation in dispersal is purely stochastic and unrelated to species- or allele-identity; such forecasting uses *q =* 2 for species assemblages [18,42] and *q =* 0,1,2 for genetic variants within a species [5]. This somewhat surprising result is consistent with findings that individuals, even of very different species, might have their dispersal more affected by gross physical effects such as currents and winds, than by their individual locomotion ability [47]. This also agrees with empirical and modeling results which indicate that geographic connectivity might be less affected by dispersal ability of particular types than by the relative reproductive output of the types [48,49]; relative reproductive output is discussed under adaptation below. Despite the success of forecasting when assuming that all types disperse equally, it is likely that forecasting will sometimes be improved by adding differential dispersal of different species or allelic types. Such forecasting may be developed from the mutual information *q =* 1 methods above, given their good performance in the simpler case of equal chance of dispersal for all types [5].

Adaptation, central to both ecology and evolution, has been addressed by a variety of entropic methods (Table 2). Note that for both molecular and species variants, there can be processes that eliminate one type in favor of another (“directional selection” in Table 2), or other processes that actively maintain more than one type (“balancing selection” in Table 2). There has been some success in modeling ecological assemblages without assessing adaptive differences between species [18]. However, there are now moves to make models that include adaptive differences between guilds of species [50]. Frank [13] and Day [12] have made a very clear case for assessing biological adaptation by entropic methods, which are a general method that allows us to connect underlying causes—such as adaptive differences of variants—to the resulting macropatterns, such as diversity within and between locations. For example, survival of individuals of a particular type (alleles, species) must often be combined over different life-stages such as:

’survival birth to juvenile (e.g., 0.4 chance of survival)’, then…‘survival juvenile to breeder (e.g., 0.6 survival)’

so that multiplication of the successive chances of survival, to give overall survival from newborn to adult breeder

‘survival from newborn to breeder = (0.4 × 0.6)’

is equivalent to addition of the logs of the survivals, and thus one often uses log fitnesses, e.g., log (*p*’/*p*), where *p* is the proportion of a particular type before selection and *p’* is its proportion after selection. Then the average of the log fitnesses is

$$\;\mathrm{Average}\;\mathrm{fitness}=\;\underset{p}{\sum }p\log\left(p\prime /p\right)=\;-KL\;$$

where *KL* is the classic expression for relative entropy (Kullback–Liebler) of the adult array of types relative to the initial newborn array [13]. This calculation provides immediate access to the maximum entropy production approach that is widely used throughout science for exploiting hypotheses about fundamental processes (e.g., inheritance mode and dispersal) to create forecasts of measurable patterns, including ecological adaptation and assemblages [51,52,53,54,55] (although some of those are not based on the four fundamental processes outlined above [51]). Analysis of adaptation might also exploit the similarity of Kullback–Liebler to logit methods already used for analysis of adaptation [5]. 

Moreover, many tests for traits that are important in adaptation rely upon contrasts between variation within and between localities. For example, if selection is in different directions in two localities, one expects to see different arrays of species or alleles, whereas if there is the same selection in all areas, one expects uniformity. Therefore, many tests for adaptation compare the amount of variation within (α) and between (β) locations [56,57,58]. Such tests can benefit from many of the essential features of Shannon (*q =* 1) such as the complete independence of within- and between-group measures, which is not easily achieved with the more commonly used *q =* 2 methods [5]. Finally, functional differences of variants (such as alleles or species) are obviously crucial to adaptation, and there are now methods for incorporating functional divergence for measures based on any *q*-value, without violating fundamental properties of diversity measures [59].

The unfilled areas in Table 2 mostly involve more than one variant (e.g., multiple species or multiple locations in the genome), AND more than one locality, AND adaptation—a very realistic and important situation! Of course, this quite complex situation is challenging for all values of *q*. However, for *q =* 1, we can anticipate that further developments will benefit from the special properties of *q =* 1 discussed earlier in this subsection, especially those properties that facilitate analysis of adaptation, dispersal, and divergence.

## 4. Beyond Ecology and Evolution

The whole of biology is fundamental to ecology and evolution. For example, perhaps the single most important common process, adaptation, is underpinned by the cell- and molecular-biology that produce the phenotype (together with ecological influences). Of course, the phenotype is the critical link between inheritance and ecological pressures, thus creating the interactions that result in natural selection and adaptation. Likewise, the nervous system is molded by evolution, and drives behavior, which is crucial to ecology and evolution. This section deals briefly with such aspects of biological information and entropy, then the next section extends this to show links with non-biological aspects of information.

As well as the innovation methods mentioned in previous sections, ecology and evolution are both heavily affected by other types of innovation, such as behavioral innovations, based on either adaptive responses within nervous systems, or remodeling of the nervous system by evolution of molecular information; the connection between these different aspects of biological information has been expressed in entropic terms [60].

Transmission and replication can also be broadened, to include not only inheritance, but other information processes such as nerve transmission and learning. Taking this broader approach, transmission of all types biological information goes beyond what is explained in Table 2, having three fundamental replication modes, with different entropic implications [43]:

- The simple type seen with cells within individuals, or individuals within a population or ecological assemblage, having an exponential rate equation,
- the autocatalytic type seen with some macromolecules, having a hyperbolic rate equation and,
- the template-dependent type, as seen with nucleic acids, having a parabolic rate equation.

The different rate equations for these processes are further modified by density, competition for space, energy, and resources, etc., as well as showing considerable stochasticity. Some replicators have become dependent upon others; for example, many nucleic acids only replicate as a synchronous part of a cell replication cycle that has a fundamentally different rate equation, which itself is often constrained within replicating individuals [43]. In contrast, other molecules are partly independent of the cell cycle, including viruses, epigenetic modifications, and prions. Nerve impulses might show any of these three replication modes, depending upon the way the nerve network is connected. The same is true for behavioral transmission such as learning in populations with differently configured social networks.

Broadly speaking, adaptation includes not only selection, but interaction with all other information processes such as behavioral avoidance of harm [60] or molecular interactions. Thus adaptation requires modeling and assessment of physical and functional networks of heritable information. There is already extensive use of Shannon-based methods for expressing associations within networks of genes that are interacting either by physical linkage, or through expression pathways [5,17,37,61].

## 5. Extended Ecology and Evolution

The four basic processes are found beyond ecology, likely including prebiotic transmission and prebiotic adaptation to the physical environment or competition [62]. Moreover, biological information has continuously sprouted offshoots such as the nervous system, electronic information systems, etc. Every issue of the journal *Entropy* attests that information approaches apply well to innovation, transmission, adaptation, and movement in the physical world. Again, these processes can be expressed as probabilities of alternatives, such as SNP alleles or the 0 versus 1 for a binary string in computing. As a result, there is much borrowing of mathematical approaches, not only within biology [5], but also between genetic theory and computer algorithm design [63,64].

Perhaps even more powerful might be to consider one continuous process that encompasses innovation, transmission, adaptation, and movement, from the prebiotic physical environment [43], through biology, to the physical environment including modern information technology applications (Table 3). These different systems interact strongly, often being dependent upon one another, over various time-scales. For example, within nervous systems, rapid innovation of impulses and connections is limited by the broad architecture of the network, which ultimately derives from slow DNA or epigenetic changes taking place over a longer time-scale. Also, information technology is still dependent upon our biological neuronal systems to build and program machines.

Evolutionary algorithms are modeled on the same four processes of biological evolution, and are used to explore for potentially improved computer code [63,64]. These algorithms usually mimic only some aspects of biological evolution, such as mutation, recombination, selection, and associative overdominance [68]. In the latter, advantageous or disadvantageous code affects the transmission of nearby code that is selectively neutral. The progress of associative overdominance depends upon the combination of selective advantage/disadvantage, and the rate at which parts of the code are swapped between scripts—the mimic of recombination [69]. There are other areas where biology and evolutionary algorithms converge, such as genetic ‘diploid’ or ‘polyploid’ code, which is a form of what is called parallelism in computing: each biological individual has two or more slightly different versions of the genome, and sometimes individuals with two (or more) versions perform better, which is a type of ‘balancing’ selection that maintains variation. For both biology and evolutionary algorithms, there is an enormous array of possible novelties, called the ‘adaptive landscape’, so exploring these possibilities requires systematic methods, which are highly developed in phylogenetics and other aspects of biology [64,69,70]. The problem of exploring a huge space of molecular interactions has been extensively investigated with *q =* 1 methods, sometimes with great success in medical genetics and molecular biology [15,16,71]. 

The interaction between evolutionary algorithms and artificial intelligence extends beyond their shared mathematics. First, just as the nervous system’s information arose out of heritable information such as DNA, our nervous systems’ information has given rise to evolutionary algorithms, and one of their manifestations, artificial intelligence (AI). Secondly, the nervous system can lead particular individuals to move to places where their heritable information makes them better adapted, such as moving a cold-sensitive individual to a warmer place, where it might survive and reproduce better. There is no reason why artificial intelligence should not result in such adaptive behavior of both living organisms and nonliving mechanisms. Indeed there is great interest in using AI to understand (and therefore manipulate?) the behavior of neuron networks, as well as group decisions by an ‘intelligent swarm’ of humans [66], so that all the systems in Table 3 interact extensively as part of a continuum of information. Any value of *q* might help in these applications, but we might see special utility for *q =* 1 biological theory, because of its good performance at tracking and forecasting each of the four processes, as outlined in Table 1 and Table 2, as well as the utility of *q =* 1 for exploring a huge space of alternatives.

**Figure 2:**
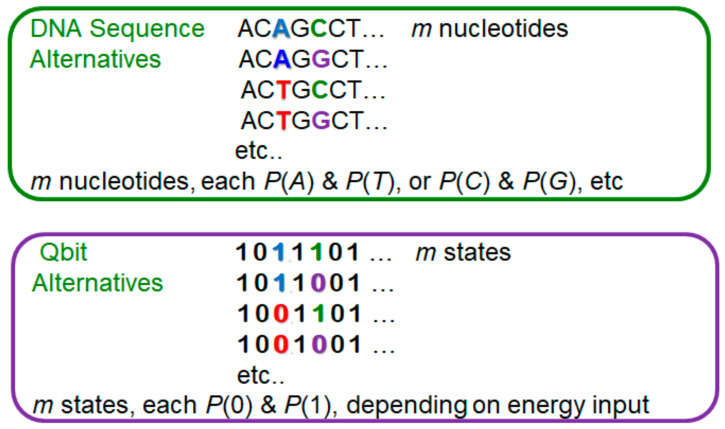
Similarities of DNA and Quantum Computing. In the DNA in the upper panel, if association between individual SNPs is random (‘linkage equilibrium’), then the proportion of a particular DNA sequence (‘haplotype’) is the product of the proportions at each SNP in the population, over *m* nucleotide positions. Similarly, for the parallel quantum ‘qbits’ in the lower panel, each will have a probability of being zero or 1, depending upon the input of energy to that part of the quantum computer (which affects the complex amplitude, whose square is the probability). Like the DNA sequence, the expected outcome in a quantum computer would be characterized by the product of the *m* probabilities, P.

It is likely that the similarities of biological evolution and evolutionary algorithms will become more noticeable when quantum computing becomes a day-to-day reality [60,66]. This is because of the probabilistic and parallel nature of quantum computing mimics biology closely. First, the behavior of qbits is stochastic, collapsing, upon observation, to one state or another with probabilities determined by the prior input of energy to that qbit [63,72]. Second, it is said that massive parallelism will be important for efficient quantum computing [63,72]. The result is that quantum computing displays some close similarities to a process called balancing selection in biology, where two allelic states are maintained in a population (equivalent to the computer parallelism), with their relative frequencies maintained by selective forces that act against individuals that contain only one type of allele. In stochastic genetic systems, this situation has the counterintuitive behavior that if the expected equilibrium proportions are near the absorbing boundaries—0 or 1—then the forces that would be expected to maintain both variants actually increase the chance of losing one of the variants [73,74]. In future, this behavior may also occur in quantum computing. Again, Shannon’s utility in assessing selection might be useful for quantum computing, just as for evolutionary computing. Figure 2 shows an example of analogy between DNA nucleotides and qbits, in cases where there is independence within each system, i.e., no linkage of DNA nucleotides and parallelism of qbits. As described above, there are already extensive methods to deal with the cases where DNA nucleotides are not independent (i.e., “linked”), which can also happen with qbits.

## 6. Conclusions

Inspired by projects aiming to systematically amass all genomic information throughout life [75], it seems that modeling and understanding of information will be best served by considering a single process encompassing all evolution from prebiotic to biological-evolution to evolutionary computing. Throughout this continuum, the common information processes are Innovation, Transmission, Adaptation, and Movement. In arriving at a unified treatment of these processes, there appears to be great promise in using the new theoretical base for Shannon Entropy/Information *q* *=* 1. However, this theory needs further extension, especially to multiple locations with adaptation.

## Figures and Tables

**Table 1:** Ecological and evolutionary information or entropy, for values *q =* 0, 1, 2. (a) Measurement and (b) forecasting from underlying processes. Full equations are found in the supplement of a previous review [5].

Entropy Hq Effective Number Dq	ECOLOGY: Variant Species in an Assemblage	EVOLUTION: Variant Molecules (Genes) within Species
**(a) Measurement**		
H0 = Count of types − 1 D0 = Count of types	Used, but has very wide confidence limits, even with modern corrections [9,10].	
H1=∑plnp D1= eH1 Where *p* values are the proportions of the different variants	The most common frequency-sensitive measure [11].	Rarely used until recently [5]. Related measures are proposed as a primary measure of evolvability [12,13]. Commonly used for analyzing networks of physically linked or functionally interacting genes [5,14,15,16,17].
H2=1−Σp2 D2= 1/(1−H2)	Some use [18]	The most common measure (Heterozygosity, Nucleotide diversity, STRUCTURE, AMOVA, *F_ST_, G*”*_ST_*, *D_EST_*, etc.) [19,20,21,22,23,24].
**(b) Forecasts from Underlying Processes**		
H0 = Count of types − 1 D0 = Count of types	No forecasts from underlying processes; some from curve-fitting [25,26].	Some forecasts, with underlying transmission and innovation only [27].
H1=∑plnp D1= eH1	Forecasts are available to be transferred from Molecular Ecology [5].	Forecasting ability now close to matching that for *q =* 2 [5]. Further details are in Table 2.
H2=1−Σp2 D2= 1/(1−H2)	Some forecasts transferred from Molecular Ecology, but only with underlying transmission and innovation, no adaptation [18].	Extensive ability to forecast under a wide range of conditions for all underlying processes: Innovation, Transmission, Movement, and Adaptation. Forecasts are often based on gas diffusion theory, e.g., Fokker–Planck Equation (see summaries in textbooks [19,20])

**Table 2:** Types of forecasts available for *q =* 1 (Shannon) entropy/information, showing how they can be used for the common processes: Innovation, Transmission, Movement, and Adaptation. Although much of this modeling has been done for molecular variants, it has often been, or could be, applied to variant species in ecological assemblages, as described in Vellend (2016) [1] and text of Section 3. For forecasts with other values of *q*, see Table 1b.

UNDERLYING PROCESSES	Space and Time Scales			
	α Within-Locality		β between-Locality	
	Finite Size, at Equilibrium	Dynamic: Non-Equilibrium	Finite Size, at Equilibrium	Dynamic: Non-Equilibrium
**INNOVATION**	Innovation mechanisms—SNP, IAM and SMM—are defined and described further in the text, including the relationships between forecasts for molecules within one species (described in this table) and forecasts for species in assemblages			
**TRANSMISSION** Neutral variants (i.e., no effect on adaptation) with stochasticity	SNP [34] IAM [33,35] SMM [33,35]	SNP, IAM, SMM [36] SNP [34]	SNP [34] IAM [33,35] SMM [33,35]	SNP [34]
**MOVEMENT** Neutral variants, with dispersal between locations	-	-	SNP [34] IAM [33,35] SMM [33,35]	SNP [34]
**ADAPTATION** Continuous heritable variants, e.g., reproductive rate or gene expression patterns	[5,37,38]	[5,37,38]	Not Yet	Not Yet
**ADAPTATION** Discrete heritable variants, e.g., DNA alleles or haplotypes	‘Balancing’ selection that maintains more than one variant [39]	‘Directional’ selection that favors a single variant ([12,13] and Supp. S4, S5 of review [5])	Not Yet	Not Yet

**Table 3:** Processes common to all systems of evolution, and their likely timescales.

System	Common Processes for Information			
	Innovation	Transmission	Adaptation	Movement
Prebiotic (may be continuing slowly in current physical environment)	Many years? [65]	Seconds, or longer, rate depends upon type of interactions [43]	Speed would depend upon relative rates of innovation and competitive interactions [62].	Probably occurs, at least involuntarily in currents, etc.
Biomolecules—acting individually	Seconds, or longer	Seconds, or longer, rate depends upon type of interactions [43]	Seconds, or longer	Seconds, or longer
Biomolecules—as basis of biological evolution	Generations [19,20]	Generations [19,20]	Generations [19,20]	Generations [19,20]
Neural networks and Behavioral responses driven by neurons	Seconds	Seconds	Seconds (or longer with a group of individuals [66]	Seconds, or longer
Species	Usually 1000’s of generations [1,18,40]	Usually 1000’s of generations [1,18,40]	Usually 1000’s of generations [1,40]	Usually 1000’s of generations [1,18,40]
Algorithms and machines	Seconds to Hours [63,64,67]	Seconds to Hours [63,64,67]	Seconds to Hours [63,64,67]	Seconds to Hours e.g., Self-driving cars, Mars rovers, Computer viruses
